# A Rapid Fluorescence-Based Microplate Assay to Investigate the Interaction of Membrane Active Antimicrobial Peptides with Whole Gram-Positive Bacteria

**DOI:** 10.3390/antibiotics9020092

**Published:** 2020-02-19

**Authors:** Gerard Boix-Lemonche, Maria Lekka, Barbara Skerlavaj

**Affiliations:** 1 Department of Medicine, University of Udine, Piazzale Kolbe, 4, 33100 Udine, Italy; 2 Polytechnic Department of Engineering and Architecture, University of Udine, Via delle Scienze 206, 33100 Udine, Italy

**Keywords:** membrane depolarization, membrane permeabilization, *Staphylococcus* spp., antimicrobial peptides, potentiometric dye, propidium iodide, fluorescence-based assay, microplate reader, FE-SEM

## Abstract

Background: Membrane-active antimicrobial peptides (AMPs) are interesting candidates for the development of novel antimicrobials. Although their effects were extensively investigated in model membrane systems, interactions of AMPs with living microbial membranes are less known due to their complexity. The aim of the present study was to develop a rapid fluorescence-based microplate assay to analyze the membrane effects of AMPs in whole *Staphylococcus aureus* and *Staphylococcus epidermidis*. Methods: Bacteria were exposed to bactericidal and sub-inhibitory concentrations of two membrane-active AMPs in the presence of the potential-sensitive dye 3,3′-dipropylthiadicarbocyanine iodide (diSC_3_(5)) and the DNA staining dye propidium iodide (PI), to simultaneously monitor and possibly distinguish membrane depolarization and membrane permeabilization. Results: The ion channel-forming gramicidin D induced a rapid increase of diSC_3_(5), but not PI fluorescence, with slower kinetics at descending peptide concentrations, confirming killing due to membrane depolarization. The pore-forming melittin, at sub-MIC and bactericidal concentrations, caused, respectively, an increase of PI fluorescence in one or both dyes simultaneously, suggesting membrane permeabilization as a key event. Conclusions: This assay allowed the distinction between specific membrane effects, and it could be applied in the mode of action studies as well as in the screening of novel membrane-active AMPs.

## 1. Introduction

The World Health Organization (WHO) identified antibiotic-resistant bacteria as one of the greatest threats to human health in the future [1]. There is an urgent need for novel antimicrobial agents acting on thus far unexploited targets. Several antimicrobial peptides (AMPs) have been proposed as potential candidates for the development of novel antimicrobials [2,3,4]. Among them, those exerting their bactericidal action by membrane permeabilization are particularly interesting [2,4]. The cytoplasmic membrane of bacteria may be regarded as a valid target for at least two reasons: (i) it is essential because it is the site where processes crucial for bacterial survival take place, and (ii) it is a conserved non-protein structure which cannot be easily modified without the risk of losing its functional and structural integrity, making the emergence of resistance less likely [5]. In addition, the cytoplasmic membrane could be targeted also in dormant, quiescent cells such as those forming biofilms [6]. In fact, the membrane-targeting AMPs are bactericidal and have generally broad-spectrum activity also including biofilms [7,8].

In the past decades, membrane interactions of AMPs have been extensively investigated by using model membrane systems, and on this basis, several models of peptide-membrane interaction have been proposed [4,9,10,11]. However, due to the complexity of living microbial membranes [12,13,14], interactions of AMPs with whole bacteria may occur in a different way and possibly lead to different consequences [15,16,17]. In almost all species the cytoplasmic membrane is surrounded by a peptidoglycan layer, which is particularly thick in Gram-positive bacteria [13], but it is usually freely permeable to AMPs. On the contrary, the outer membrane in the external envelope of Gram-negative microorganisms with its peculiar lipid composition [18] represents an important permeability barrier [19], which can, however, be overcome by many AMPs [3]. Of course, all the processes related to peptidoglycan and/or lipopolysaccharide metabolisms require structural contiguity and functional interconnections with the cytoplasm through the plasma membrane [20,21,22]. Hence, events occurring at the bacterial cytoplasmic membrane, or at other levels of the external envelope, may have important repercussions on the cell interior [20,23], and vice versa the inhibition of vital intracellular processes can drastically modify membrane integrity [17]. Membrane-targeting AMPs can affect membrane functions by increasing the membrane permeability to small ions or to larger molecules, or by causing large-scale membrane damage [15,16,24,25]. To analyze the mode of action of membrane-targeting AMPs, membrane-impermeable fluorescent dyes such as propidium iodide (PI) [26] or SYTOX green [27] are frequently employed. Their fluorescence increases upon their binding to nucleic acids, which happens when membrane integrity is critically damaged or large pores are formed [27]. For this reason, they are also used to discriminate between viable and dead bacteria [26]. These dyes, however, are not suitable to detect modifications in membrane permeability to small ions, which may be lethal to bacteria also in the absence of evident membrane lesions [28,29]. The modifications in ion permeability could be studied using membrane potential-sensitive fluorescent probes such as the oxonol bis-(1,3-dibutylbarbituric acid)trimethine oxonol (DiBAC_4_(3)) [30], or carbocyanine dyes such as 3,3′-dihexyloxacarbocyanine iodide (DiOC_6_(3)) [31] and 3,3′-dipropylthiadicarbocyanine iodide (diSC_3_(5)) [32]. These are lipophilic dyes, with the difference that, at physiological pH oxonols are anionic, while carbocyanines carry a positive charge [33]. This implies that DiBAC_4_(3) cannot penetrate the polarized membrane of living microorganisms, and only enters depolarized cells, displaying enhanced fluorescence and red spectral shift upon binding to intracellular proteins [26,34]. On the other hand, carbocyanines such as diSC_3_(5) accumulate on polarized membranes resulting in self-quenching of fluorescence [35,36]. Upon membrane depolarization, the dye is released and fluorescence de-quenched. In many studies, these dyes were used alone or in combination with PI, as a means to evaluate bacterial viability [37,38], antibiotic susceptibility [39,40], and to monitor the physiological state of individual microbial cells [41] by flow cytometry. Furthermore, these dyes were applied in mode-of-action studies to specifically analyze membrane depolarization of Gram-positive [28,42,43] and Gram-negative [32,44,45] microorganisms upon treatment by different AMPs, or other membrane-active compounds [29,46]. In these studies, the fluorescence was measured by flow cytometry [42], spectrofluorimetry by using cuvettes [28,32,43,44,45] or microplates [29,46]. An advantage of using a microplate reader, equipped with the temperature control system, is the possibility to follow the changes in fluorescence kinetically and, in addition, to monitor several samples simultaneously. Both, the oxonol DiBAC_4_(3) and the carbocyanine dye diSC_3_(5) have been proposed for kinetic monitoring of changes in membrane polarity of Gram-positive bacteria exposed to antimicrobial compounds [34,47].

In the present study, we partially adapted these latter methods to two Gram-positive species representing important human pathogens, namely *Staphylococcus aureus* and *Staphylococcus epidermidis*. *S. aureus* has been ranked by WHO as a high priority pathogen for the development of new antibiotics, mainly due to the high prevalence of methicillin-resistance and increasing resistance to vancomycin [48]. *S. epidermidis* represents an emerging opportunistic pathogen responsible for foreign-body associated infections due to its ability to form biofilm [49]. Hence, both species may be considered as suitable targets for the set-up of a novel screening method of membrane-active compounds effective against Gram-positive microorganisms. Moreover, from a practical point of view, as their envelope is devoid of the outer membrane, the Gram-positives have the technical advantage that there is no need for any preliminary permeabilization step, avoiding the risk of artefactual results [31]. The aim of the present study was to develop a rapid fluorescence-based microplate assay to get mechanistic and kinetic insights into the interaction of membrane-active AMPs, such as the ion channel-forming gramicidin D [50], and the pore-forming melittin [51], with cytoplasmic membranes of whole *S. epidermidis* and *S. aureus*. By combining the potentiometric dye diSC_3_(5) with the DNA-staining dye PI, it was possible to simultaneously monitor the peptide-induced phenomena of membrane depolarization due to ion movements across the membrane, and membrane permeabilization due to the formation of pores, and thus to distinguish between specific membrane effects due to different modes of peptide-membrane interaction.

## 2. Results

### 2.1. Compatibility between the Fluorescent Dyes

**Figure 1:**
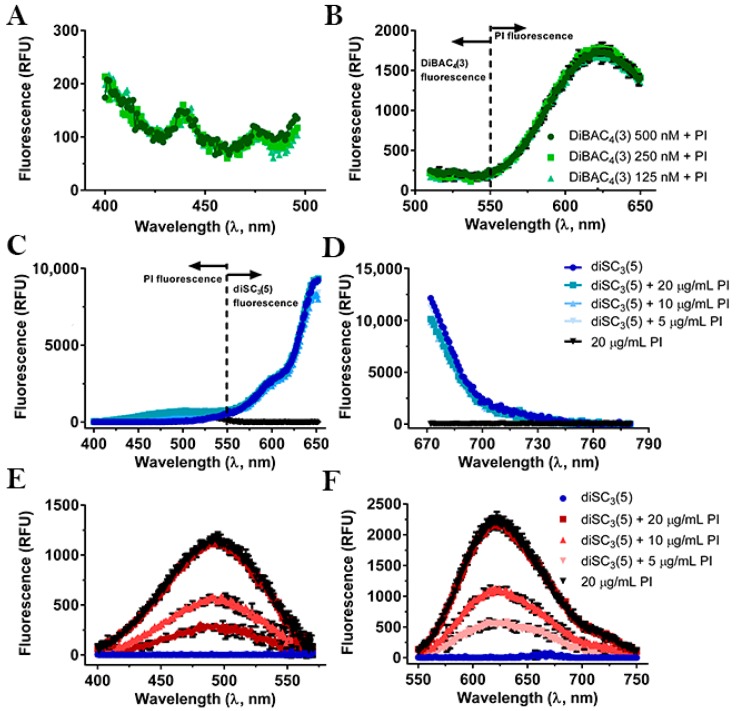
Fluorescence excitation (**A**) and emission (**B**) spectra of bis-(1,3-dibutylbarbituric acid)trimethine oxonol (DiBAC_4_(3)) + Propidium Iodide (PI) and fluorescence excitation (**C**,**E**) and emission (**D**,**F**) spectra of 3,3′-dipropylthiadicarbocyanine iodide (diSC_3_(5)) +/− PI. (**A**) Excitation spectra with 516 nm detection wavelength (DiBAC_4_(3) emission maximum); (**B**) emission spectra with 496 nm excitation wavelength (DiBAC_4_(3) excitation maximum) of three different DiBAC_4_(3) concentrations + 10 µg/mL PI; (**C,E**) excitation spectra with 672 nm (diSC_3_(5) emission maximum) and 617 nm (PI emission maximum) detection wavelengths, respectively, and (**D,F**) emission spectra with 652 nm (diSC_3_(5) excitation maximum) and 490 nm (PI excitation maximum) excitation wavelengths, respectively, of 400 nM diSC_3_(5) + 20, 10, and 5 µg/mL PI. For technical reasons due to instrument settings, spectra in (**A**–**D**) could not be measured at wavelengths >496 nm, <510 nm, >650 nm, and <670 nm, respectively. In (**A**), (**C**), and (**D**) error bars were omitted for clarity.

The spectroscopic properties of two potentiometric dyes, the oxonol DiBAC_4_(3) and the carbocyanine diSC_3_(5), and of the DNA stain PI, were evaluated in our assay conditions by measuring fluorescence with a microplate reader by using the reported wavelength values for excitation and emission maxima [30,35,52,53,54]. These values were confirmed when spectra were measured separately (see Supplementary Figures S1 and S2). However, as evident from Figure 1, when DiBAC_4_(3) was combined with PI, we observed strong interference of PI towards DiBAC_4_(3) (Figure 1A,B). This finding precluded the use of the combination DiBAC_4_(3) + PI in our fluorescence assay. On the other hand, the fluorescence of diSC_3_(5) was not affected by PI (Figure 1C,D) and vice versa (Figure 1E,F), thus allowing their combined use for our purposes.

### 2.2. Selection of Membrane-Active Peptides

The next step was to find the antimicrobial peptide (AMPs) candidates as positive controls for membrane depolarization and/or membrane permeabilization events. Based on scientific literature, four membrane-active AMPs were selected: gramicidin D, cecropin A, magainin 2, and melittin. The insect peptide cecropin A [55], the frog peptide magainin 2 [56], and the cytolytic peptide melittin from bee venom [51], are all cationic α-helical AMPs known to permeabilize bacterial membranes [57]. Melittin, in particular, is well-known as a pore-forming peptide [11]. Gramicidin D is a bacterial antibiotic produced by *Bacillus brevis* [58,59]. It is composed of a mixture of highly similar pentadecapeptides consisting of about 85% gramicidin A [50]. The principal structural features of this peptide are alternating hydrophobic L- and D-amino acid residues and their organization in the membrane environment leads to the formation of an ion channel [50]. The antimicrobial activity of these peptides was evaluated against representative Gram-positive and Gram-negative bacterial species in a standard minimum inhibitory concentration (MIC) assay. Results in Table 1 show that only melittin and gramicidin D displayed activities against *S. aureus* and *S. epidermidis*, which are the principal target of the present study. These two peptides were thus selected for further experiments.

### 2.3. Interference of Peptides and Uncouplers with the Fluorescent Dyes

**Figure 2:**
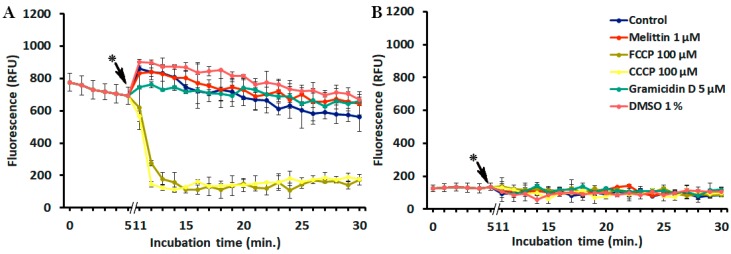
Interference of uncouplers and peptides with the fluorescent dyes. The kinetics of (**A**) diSC_3_(5) (λ_ex_ = 652 nm, λ_em_ = 672 nm) and (**B**) PI (λ_ex_ = 535 nm, λ_em_ = 617 nm) are shown in separate graphs for clarity purposes. The time necessary for the addition of peptides and uncouplers (indicated by *) was about 6 min.

To verify whether the selected peptides could affect the fluorescence of diSC_3_(5) or PI, the fluorescence of both probes combined together was monitored kinetically in the presence of each AMP. Two widely used uncouplers, i.e., carbonyl cyanide 3-chlorophenylhydrazone (CCCP) and carbonyl cyanide 4-(trifluoromethoxy)phenylhydrazone (FCCP), to be possibly used as controls of complete depolarization, were also included in these experiments. As shown in Figure 2, both uncouplers decreased diSC_3_(5) fluorescence, in line with the report of te Winkel et al. [34], whereas the peptides did not. No interference between PI and the added molecules was recorded. Unfortunately, the interference of the uncouplers with diSC_3_(5) fluorescence precluded their use in this assay.

### 2.4. Monitoring of Peptide-Induced Membrane Alterations with the Combination of diSC_3_(5) and PI

To monitor the phenomena of membrane depolarization and membrane permeabilization simultaneously, *S. epidermidis* ATCC 35984 or *S. aureus* ATCC 25923 were incubated with the ion channel-forming gramicidin D, or with the pore-forming melittin, in PBS-glc containing 400 nM diSC_3_(5) and 5 µg/mL PI at 37 °C in 96-well black microtiter plates (Optiplate, PerkinElmer, Waltham, MA, USA). Fluorescence was measured throughout the assay and, at 30 min incubation, aliquots were taken to determine bacterial viability by colony forming units (CFU) counts.

**Figure 3:**
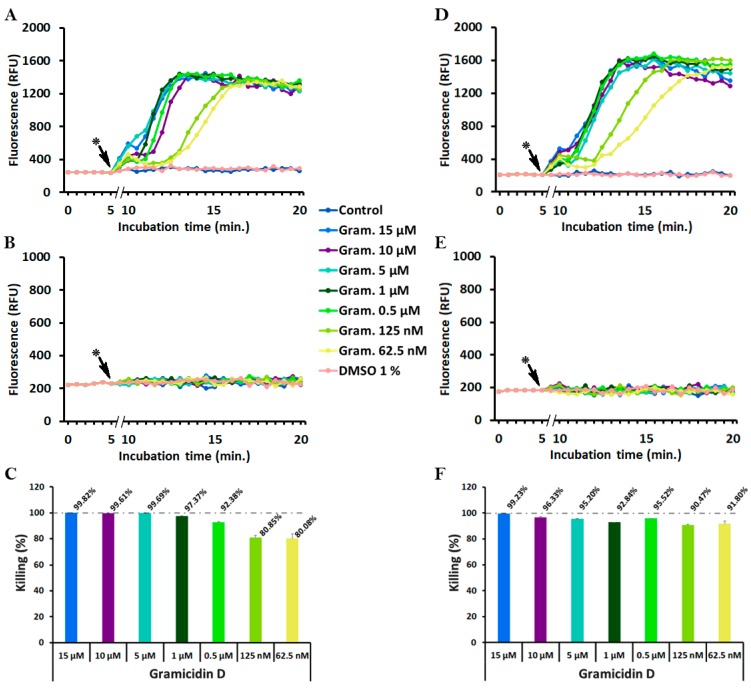
Membrane depolarization (diSC_3_(5)) (**A**,**D**), permeabilization (PI) (**B**,**E**) and killing (**C**,**F**) of *S. epidermidis* (**A–C**) and *S. aureus* (**D–F**) caused by gramicidin D. Experiments were performed with 10^8^ CFU/mL of the indicated strains in PBS-glc with 400 nM diSC_3_(5) (λ_ex_ = 652 nm, λ_em_ = 672 nm) and 5 µg/mL PI (λ_ex_ = 535 nm, λ_em_ = 617 nm) at 37 °C. CFU counts were determined at 30 min incubation. For clarity purposes diSC_3_(5) and PI kinetics are in separate graphs, error bars were omitted and only the initial 20′ are shown. (*) peptide addition.

Figure 3 shows that in both *Staphylococcus* spp. gramicidin D induced a significant increase of diSC_3_(5) (Figure 3A,D), but not of PI fluorescence (Figure 3B,E). The increase was rapid at bactericidal concentrations (0.5–15 μM, >92% killing (Figure 3C,F)), while it slowed down, with more sigmoidal shapes, at descending peptide concentrations (62.5–125 nM) that also produced lower killing (80%). Remarkably, all curves reached more or less the same maximum fluorescence values, although with different slopes and thus, at different times. There was a clear correlation between gramicidin D concentrations, bacterial killing, and diSC_3_(5) fluorescence kinetics (Figure 3A,C,D,F), whereas in none of the cases PI fluorescence increased (Figure 3B,E). In addition, it is important to note that diSC_3_(5) and PI per se were not toxic to bacteria because in the absence of gramicidin D neither of the two probes showed an increase of fluorescence (Figure 3A,B,D,E), or caused a decrease of CFUs (data not shown).

**Figure 4:**
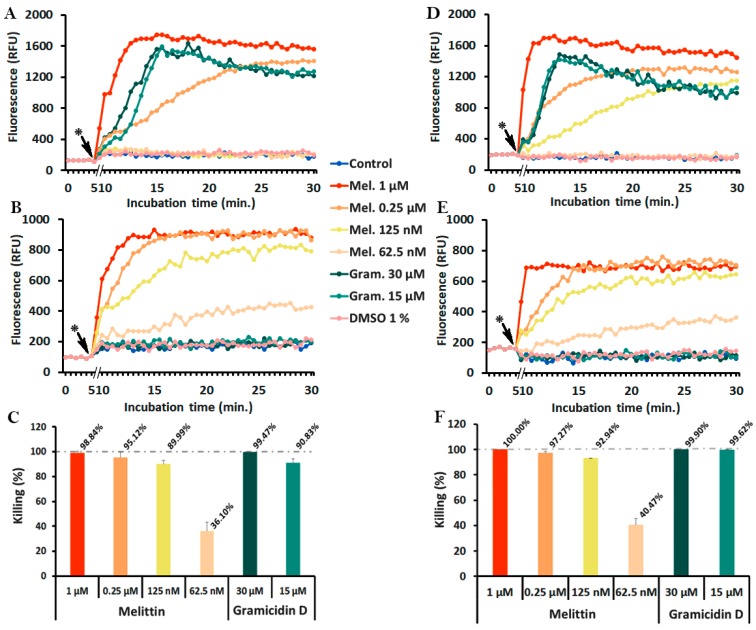
Membrane depolarization (diSC_3_(5)) (**A**,**D**), permeabilization (PI) (**B**,**E**) and killing (**C**,**F**) of *S. epidermidis* (**A–C**) and *S. aureus* (**D–F**) caused by melittin and gramicidin D. Experiments were performed with 10^8^ CFU/mL of the indicated strains in PBS-glc containing 400 nM diSC_3_(5) (λ_ex_ = 652 nm, λ_em_ = 672 nm) and 5 µg/mL PI (λ_ex_ = 535 nm, λ_em_ = 617 nm) at 37 °C. CFU counts were determined at 30 min incubation. The kinetics of diSC_3_(5) and PI are shown in separate graphs and error bars were omitted for clarity. (*) peptide addition.

The results obtained with melittin gave a completely different picture (Figure 4). This pore-forming peptide caused a remarkable, dose-dependent increase of PI fluorescence (Figure 4B,E), with a slower increase at sub-MIC (62.5–125 nM) and more rapid kinetics at bactericidal concentrations (0.25–1 μM, killing >95%) (Figure 4C,F). DiSC_3_(5) fluorescence increased only at bactericidal melittin concentrations (0.25–1 μM) (Figure 4A,D).

### 2.5. Morphology of Peptide-Treated S. epidermidis

**Figure 5:**
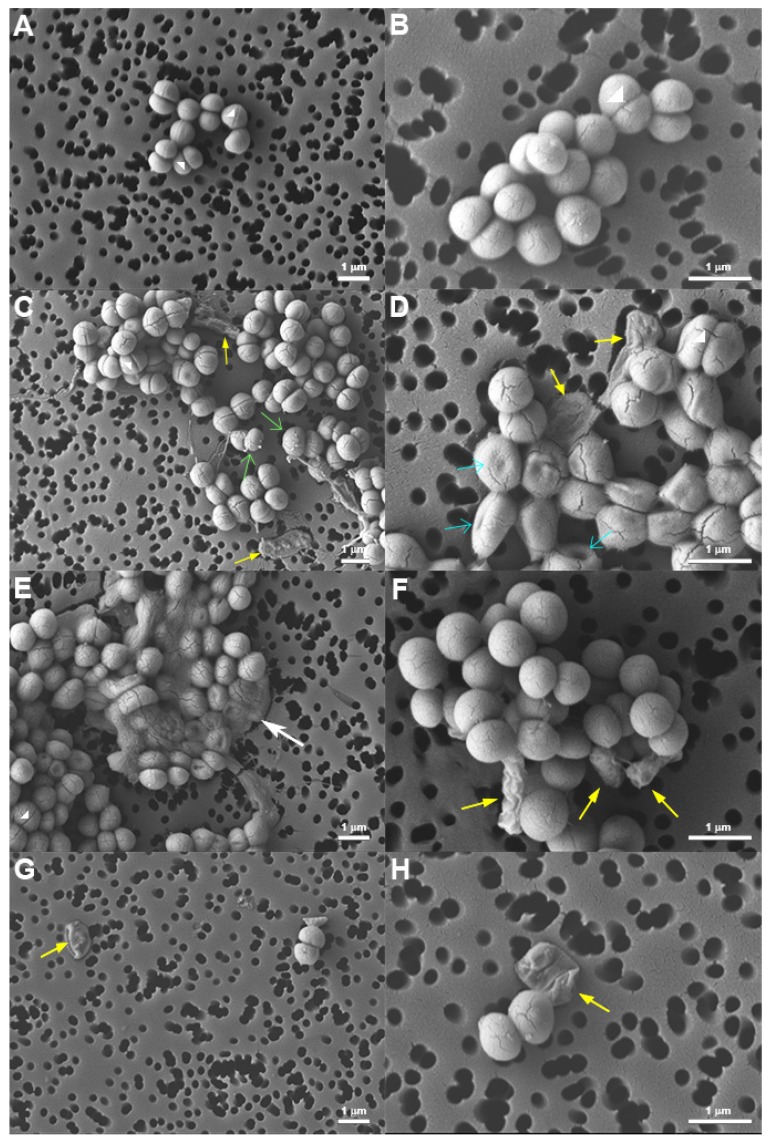
Morphology of *S. epidermidis,* untreated (**A**,**B**) or treated with 30 µM gramicidin D (**C**,**D**), 1 µM melittin (**E**,**F**) and 125 nM melittin (**G**,**H**) analyzed by Field Emission Scanning Electron Microscopy (FE-SEM). Arrows indicate, respectively, division septa (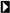), blebs (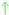), indents (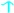), bacteria with altered morphology (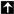), and deflated bags (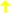). Representative images from two experiments performed in duplicate are shown.

To investigate whether the distinct membrane effects induced by melittin and gramicidin resulted in different morphology of treated bacteria, at the end of fluorescence kinetics (i.e., 30-min time point, around 20 min incubation with peptides) bacteria were processed for analysis by Field Emission Scanning Electron Microscopy (FE-SEM). The micrographs revealed clear differences in bacterial morphology and an evident decrease in bacterial density in treated samples compared to untreated controls, in line with the CFU determinations performed in parallel. Images of untreated bacteria (Figure 5A,B) show the normal, round and smooth appearance of vital and growing staphylococcal cells.

On the contrary, images of peptide-treated samples show, besides cells with normal appearance, bacteria with remarkably altered morphology. Upon treatment with gramicidin, several cells presented indents, similar to what observed by Hartmann et al. with gramicidin S [60], or looked deflated (Figure 5D). Furthermore, on the surface of many gramicidin-treated cells, there were protrusions similar to blebs. However, in some cases, these protrusions could be explained alternatively as remains of cytoplasmic material derived from lysed cells (Figure 5C). Deflated, “ghost-like” cells were observed also upon treatment with melittin (Figure 5G,H), in line with the atomic force microscopy analysis by Roncevic et al. [61]. In some cases, bacteria seemed to collapse (Figure 5E), whereas in others they appeared shriveled as raisins (Figure 5F), without macroscopic surface lesions like blebs or blisters.

## 3. Discussion

The bacterial cytoplasmic membrane represents a valid drug target for the development of novel antimicrobial agents. The interactions of membrane-active AMPs with target membranes have been extensively studied in artificial systems, usually by using a relatively high peptide-to-lipid ratio. However, knowing the complexity of living microbial membranes, there is a need for a deeper characterization of AMP-induced effects on membranes of live bacteria. Therefore, in the present study, our goal was to set up a fluorescence assay for real-time monitoring of changes in membrane permeability induced by active peptide concentrations in whole bacteria. Specifically, we focused on Gram-positive microorganisms of medical relevance such as *S. aureus* and *S. epidermidis*. For the simultaneous monitoring of membrane depolarization and membrane permeabilization, we combined the membrane-impermeable nucleic acid stain PI with a potential-sensitive dye. We wanted to understand whether these two phenomena were clearly distinct or could be causally and temporally correlated, as suggested by other authors in the case of synthetic AMPs acting on *Pseudomonas aeruginosa* [62]. Therefore, the dye combination in our assay was conceptually different from the commercially available dual stain viability kit, based on two nucleic acid stains with different membrane permeability to discriminate between live and dead bacteria [63,64].

To investigate the complex phenomena occurring at the membrane level and related to membrane integrity, bacteria should be in an optimal growth phase with fully activated oxidative metabolism. Experiments were performed with *S. aureus* and *S. epidermidis* collected at their mid-log phase, washed and resuspended in physiological salt solution supplemented with 25 mM glucose as a carbon and energy source [47]. Given the short incubation time (30 min), this condition proved fully compatible with bacterial viability, as confirmed by fluorescence measurements and CFU counts.

At difference with several published methods based on flow cytometry [65,66,67], our assay was set up in a 96-well microplate for fluorometric detection by using a microplate reader, equipped with temperature control. This allows for low assay volume, low reagent consumption, and simultaneous kinetic monitoring of multiple samples. Furthermore, by using a low-binding version of black microtiter plates (in our case OptiPlate, see Materials and Methods section) it was possible to overcome the problem of unspecific binding of hydrophobic compounds to microtiter plastic. This problem was addressed by other authors by using serum albumin in the assay mixture [34], but we preferred to avoid the use of this protein in our measurements due to possible unwanted sequestration of AMPs [68,69]. In our experience fluorescence detection with low-binding plates gave satisfactory results (see Supplementary Figure S2 for comparison between measurements of the carbocyanine diSC_3_(5) fluorescence in conventional and low-binding black microtiter plates).

The initial combination of PI with the oxonol dye DiBAC_4_(3), at difference with the report of Clementi et al. [47], was not suitable for our assay due to strong interference between the two probes, probably due to an energy transfer phenomenon similar to Fluorescence Resonance Energy Transfer (FRET) from the oxonol to PI because of spectral overlap. We next replaced the oxonol with the carbocyanine dye diSC_3_(5), which has clearly distinct fluorescence parameters and did not show any interference with PI. By using the combination diSC_3_(5) + PI we were able to simultaneously monitor the kinetics of membrane depolarization and membrane permeabilization induced by well-characterized membrane-targeting AMPs at bactericidal and sub-inhibitory concentrations. It is important to note, however, that PI fluorescence could only be detected with a sufficiently high bacterial density. In the case of the Gram-positive *S. epidermidis* and *S. aureus,* this corresponds to 10^8^ CFU/mL. We found that for the detection of both dyes together this suspension density was optimal, although a slightly lower density, i.e., 10^7^ CFU/mL, would be sufficient for the detection of diSC_3_(5) only (see Supplementary Figure S3A,B). For PI detection in the case of Gram-negative microorganisms such as *E. coli*, a bacterial density of 10^7^ CFU/mL would be sufficient thanks to their higher size and DNA content (see Supplementary Figure S3C,D).

Concerning gramicidin D, our data are in line with those reported in the literature which reveals that gramicidin D causes a rapid and full dissipation of membrane potential by forming small cation specific channels [50,59,70]. In fact, many authors used this peptide as a positive control of membrane depolarization [34,71,72]. In those studies, however, gramicidin D was used at relatively high concentrations to obtain complete depolarization, and it was often added at the end of the experiment. In our case, we focused on the suitability of our method for the detection of gramicidin-induced effects and for this reason we explored a wide range of peptide concentrations. In all cases the increase of diSC_3_(5) fluorescence reached the same level, indicating complete membrane depolarization also at lower gramicidin concentrations. In this latter case, the delay could be due to an initial compensation by the microorganism that could not be pursued over a certain point resulting in a delayed, yet lethal effect. However, PI fluorescence did increase in any of the cases, even at the highest peptide concentrations. It is important to note in this regard that the diameter of gramicidin D-induced channels is estimated to measure about ≈4 Å, what is sufficient to accommodate the passage of monovalent cations [50], but not the uptake of larger molecules such as PI (MW = 668.4 g/mol). As a consequence, the interaction of PI with nucleic acids is precluded despite bacteria dying. Our results (Figure 3) show that gramicidin D killed *S. epidermidis* and *S. aureus* by membrane depolarization without formation of larger pores that could enable PI uptake. As confirmed by CFU counts, the membrane depolarization induced by gramicidin D was the bactericidal event. Hence, any increase in PI fluorescence, recorded after several hours of incubation, as reported by some authors [73], should be rather regarded as a consequence of bacterial death. In fact, FE-SEM images showed a complex picture, including apparently intact bacteria, those with elongated shape showing some indents, others with surface blebs, and completely emptied bags. It is clear that all these modifications are representative of different dying steps that bacteria underwent upon gramicidin treatment.

In the case of melittin (Figure 4), the increase of diSC_3_(5) fluorescence did not indicate a mode of action primarily based on membrane depolarization but was rather a consequence of irreversible membrane permeabilization, which appears as the key event. Curiously, by FE-SEM analysis we rarely noticed macroscopic membrane lesions. Instead, the appearance of the deflated or emptied bag would suggest that the emptying of cell content occurred due to osmotic imbalance or, alternatively, that membrane perturbation elicited an autolytic action. Melittin has long been considered as a paradigmatic pore-forming peptide [11,74,75,76]. Taking into consideration that melittin-induced pores, with a diameter of 25–30 Å [77], are large enough to accommodate the passage of large molecules such as PI, one can reasonably expect that inorganic ion leakage may also occur. So, in principle melittin would have the ability to alter membrane potential. It is interesting to note, however, that melittin at sub-optimal concentrations induced slower PI uptake, correlated with lower killing activity, but no increase of diSC_3_(5) fluorescence (Figure 4). In addition, at sub-inhibitory concentrations the increase of PI fluorescence never reached the high values observed at bactericidal concentrations (Figure 4). This observation would suggest a limited membrane damage, probably caused by the formation of transient pores, as reported in the literature [11,74], and that bacteria could recover by maintaining the proton motive force to some extent.

The findings obtained thanks to the combination of both fluorescent dyes contribute to the advancement of our knowledge concerning the mode of action of gramicidin D and melittin. If for instance the assay would have been performed by using diSC_3_(5) only, we would conclude that both peptides, gramicidin D and melittin, act by causing membrane depolarization. On the contrary, if we had used only PI, we would speculate that gramicidin D, at the difference with melittin, did not cause membrane permeabilization and, perhaps killed bacteria by acting on some internal target(s).

Conversely, by using the combination of diSC_3_(5) and PI, we measured the phenomena of membrane depolarization and permeabilization on the same bacteria population simultaneously. This assay thus enables us to distinguish between these two phenomena and contributes to shedding light on the mode of action of membrane-active agents such as the AMPs. This study confirmed that gramicidin D causes membrane depolarization and melittin induces membrane permeabilization. We also provide additional evidence that melittin at low concentrations probably creates transient pores causing membrane permeabilization which is reversible to some extent.

We believe that this assay could be applied in investigations aimed at unraveling the mode of action of membrane-active AMPs, as well as for screening of novel compounds targeting the cytoplasmic membrane of *S. aureus* and *S. epidermidis*.

## 4. Materials and Methods

### 4.1. Equipment

Fluorometric measurements were performed with a Multimode Plate Reader (EnSpireTM 2300, PerkinElmer, Waltham, MA, USA) by using low-binding surface 96-black polystyrene microtiter plates (OptiPlate, PerkinElmer, Waltham, MA, USA ) to prevent unspecific binding of the molecules used in the study.

### 4.2. Peptides and Other Reagents

The selected membrane-active peptides gramicidin D (formyl-VGALAVVVWLWLWLWG-NHCH_2_CH_2_OH), cecropin A (KWKLFKKIEKVGQNIRDGIIKAGPAVAVVGQATQIAK-NH_2_), magainin 2 (GIGKFLHSAKKFGKAFVGEIMNS-OH), and melittin (GIGAVLKVLTTGLPALISWIKRKRQQ-NH_2_) were purchased from Sigma-Aldrich^®^ (St. Louis, MO, USA). Cecropin A and gramicidin D were dissolved in DMSO and magainin 2 and melittin in pyrogenic water. The stock solutions were kept at −20 °C. The uncouplers carbonyl cyanide 3-chlorophenylhydrazone (CCCP) and carbonyl cyanide 4-(trifluoromethoxy)phenylhydrazone (FCCP), the membrane potential-sensitive fluorescent distributional probes bis-(1,3-dibutylbarbituric acid)trimethine oxonol (DiBAC_4_(3)) and 3,3′-dipropylthiadicarbocyanine iodide (diSC_3_(5)), and the membrane-impermeable fluorescent dye propidium iodide (PI) were purchased from Sigma-Aldrich. Stock solutions were prepared as follows: 400 µM diSC_3_(5) in 100% DMSO, 50 µM DiBAC_4_(3) in 100% DMSO, and 1 mg/mL PI in ddH_2_O (PI would precipitate in a more concentrated solution). All stocks, were protected from light by aluminum foil and were stable at −20 °C for at least 6 months.

### 4.3. Bacteria and Bacterial Cultures

Two Gram-positive, *Staphylococcus epidermidis* ATCC 35984 and *Staphylococcus aureus* ATCC 25923, and two Gram-negative, *Escherichia coli* ATCC 25922 and *Pseudomonas aeruginosa* ATCC 27853 reference strains were obtained from American Type Culture Collection (ATCC; Manassas, VA, USA). Bacteria were maintained on Mueller–Hinton (MH, Difco laboratories, Detroit, MI, USA) agar plates. For antimicrobial assays, bacteria were cultured in liquid Brain Heart Infusion (BHI) overnight, 1:50-diluted in fresh medium and allowed to grow in an orbital shaker at 37 °C. Mid-log phase bacteria were harvested after 10 min centrifugation at 1000× *g* and resuspended in phosphate-buffered solution (PBS) to optimal density assessed by turbidity at 600 nm, with reference to previously determined standards. For fluorescence kinetics, mid-log phase bacteria were collected by centrifugation at 1000× *g*, washed two times with PBS (pH 7.4), and finally resuspended in PBS supplemented with 25 mM glucose (PBS-glc) at the desired density.

### 4.4. Determination of Minimum Inhibitory Concentration (MIC) and Minimum Bactericidal Concentration (MBC)

The minimum inhibitory concentration (MIC) of selected peptides was determined by a broth microdilution assay in 96-well microtiter plates, using MH broth with logarithmic-phase microorganisms at 5 × 10^5^ CFU/mL, as previously reported [68], following CLSI guidelines. The minimum bactericidal concentration (MBC) was determined by seeding aliquots from wells showing no visible growth on solid medium to allow colony counts.

### 4.5. Excitation and Emission Spectra

To verify the correct excitation and emission wavelengths of each fluorescent dye, their excitation and emission spectra were measured in our assay conditions. DiBAC_4_(3) was studied at 125, 250, and 500 nM (λ_ex_ = 496 nm, λ_em_ = 516 nm) [53] plus PI at 10 µg/mL (λ_ex_ = 490 nm, λ_em_ = 617 nm) [52] in PBS supplemented with 25 mM glucose (PBS-glc). Furthermore, the excitation and emission spectra of 400 nM diSC_3_(5) alone (λ_ex_ = 652 nm, λ_em_ = 672 nm) [53], and 400 nM diSC_3_(5) plus 5, 10, and 20 µg/mL PI, were measured in PBS-glc.

### 4.6. Interference of Uncouplers and Peptides with the Fluorescent Dyes

To verify whether uncouplers, peptides, and solvents have any interference with diSC_3_(5) or with PI, both fluorescent dyes (400 nM diSC_3_(5) and 5 µg/mL PI) combined together in the same wells of a black 96-well plate (Optiplate) were monitored kinetically for 30 minutes at 37 °C in PBS-glc in the presence of each selected molecule.

### 4.7. Kinetic Fluorescence Measurements to Detect Membrane Depolarization and Permeabilization

Mid-log phase *S. epidermidis* and *S. aureus*, resuspended at 1 × 10^8^ CFU/mL in PBS-glc (prepared as described above) were incubated in the orbital shaker at 37 °C for 15 min. Thereafter, diSC_3_(5) and PI were added at final concentrations of 400 nM and 5 µg/mL, respectively. These concentrations were established in preliminary experiments and were optimal in our conditions (data not shown). The suspension was mixed by short vortexing and 200 μL was added to the wells of a black 96-well plate (Optiplate). Samples were preincubated at 37 °C with fluorescence measurements every minute for 5 min, or until readings were stabilized. After this time, the plate was ejected. Depolarizing agents were added in duplicate wells at the desired final concentrations. The plate was rapidly placed back into the reader to continue monitoring diSC_3_(5) and PI (diSC_3_(5); λ_ex_ = 652, λ_em_ = 672 nm and PI; λ_ex_ = 535, λ_em_ = 617 nm) [54], every 0.5 min for 10–20 min. At the end of incubation (around 30 min), aliquots were withdrawn from each well, serially diluted and plated on MH agar to allow CFU determination. The killing percentage was calculated with reference to untreated samples.

### 4.8. Field Emission Scanning Electron Microscopy (FE-SEM) of S. epidermidis

The morphology of *S. epidermidis*, deposited on polycarbonate filters, were studied by Field Emission Scanning Electron Microscopy (FE-SEM) (JEOL model JSM-7610FPlus). Briefly, upon 30 min incubation, as described above, all samples were collected by centrifugation at 1000× *g* for 10 min and fixed with 2.5% (v/v) glutaraldehyde in PBS for 1 h at 4 °C. Fixed bacteria were extensively rinsed with filtered sterile PBS and dehydrated in graded series of ethanol solutions (20 min each) by centrifuging at each step. Finally, samples were deposited on 0.2 µm Isopore polycarbonate membrane filters (MerckMillipore, Burlington, MA, USA) and sputter-coated with a thin gold layer prior to FE-SEM analysis. The images were collected in the secondary electron detection mode. The working distance was set to 8 mm, the acceleration voltage to 5 keV, and the probe current to 11 to decrease the interaction depth and obtain more detailed information on the surface. FE-SEM was performed in duplicate for each sample.

## Figures and Tables

**Table 1:** Antimicrobial activities of selected antimicrobial peptides (AMPs) against reference strains.

	Cecropin A	Melittin	Magainin 2	Gramicidin D
	MIC (MBC) (µM) ^a,b^			
*S. epidermidis* ATCC 35984	>128	0.5 (0.5)	>128	2 (4)
*S. aureus* ATCC 25923	>128	0.5 (1)	64 (128)	4 (8)
*E. coli* ATCC 25922	0.75 (0.75)	1 (1)	8 (16)	>32
*P. aeruginosa* ATCC 27853	1 (2)	1 (2)	64 (64)	>32

^a^ Determined in MH broth at 5 × 10^5^ CFU/mL. ^b^ Data are means of at least 2 independent experiments.

## Supplementary Materials

The following are available online at https://www.mdpi.com/2079-6382/9/2/92/s1, Figure S1: Separate fluorescence Excitation (A,C) and Emission (B,D) spectra of bis-(1,3-dibutylbarbituric acid)trimethine oxonol (DiBAC_4_(3)) (A–B) and propidium iodide (PI) (C–D) in phosphate-buffered saline supplemented with 25 mM glucose (PBS-glc). (A,C) Excitation spectra of 500 nM DiBAC_4_(3) with 516 nm (A) and 20 µg/mL PI with 617 nm (C) detection wavelengths. (B,D) Emission spectra of 500 nM DiBAC_4_(3) with 496 nm (B) and 20 µg/mL PI with 490 nm (D) excitation wavelengths. For technical reasons due to instrument settings, spectra could not be measured at wavelengths >496 nm (A) and <510 nm (B), Figure S2: Fluorescence Excitation and Emission spectra of 400 nM nM 3,3′-dipropylthiadicarbocyanine Iodide (diSC_3_(5)) in black wellplates (A,B) and low-binding OptiPlate (C,D). (A,C) Excitation spectra with 672 nm detection wavelength, and (B,D) emission spectra with 652 nm excitation wavelength (error bars were omitted for clarity). For technical reasons due to instrument settings, spectra could not be measured at wavelengths >650 nm (A,C) and <670 nm (B,D), Figure S3: Membrane depolarization (diSC_3_(5)) (A) and Killing (B) of *S. epidermidis* ATCC 35984 caused by gramicidin D and membrane permeabilization (PI) (C) and Killing (D) of *E.coli* ATCC 25,922 caused by melittin. Experiments were performed with 10^7^ CFU/mL of the indicated strains in PBS-glc with 400 nM diSC_3_(5) (λ_ex_ = 652 nm, λ_em_ = 672 nm) and 5 µg/mL PI (λ_ex_ = 535 nm, λ_em_ = 617 nm) at 37 °C. CFU counts were determined at 30 min incubation. (*) peptide addition.

## References

[1] World Health Organisation (2019). Antimicrobial resistance. https://www.who.int/news-room/fact-sheets/detail/antimicrobial-resistance.

[2] Hancock R.E.W., Sahl H.-G. (2006). Antimicrobial and host-defense peptides as new anti-infective therapeutic strategies. Nat. Biotechnol..

[3] Yeung A.T.Y., Gellatly S.L., Hancock R.E.W. (2011). Multifunctional cationic host defence peptides and their clinical applications. Cell. Mol. Life Sci..

[4] Rončević T., Puizina J., Tossi A. (2019). Antimicrobial Peptides as Anti-Infective Agents in Pre-Post-Antibiotic Era?. Int. J. Mol. Sci..

[5] Peschel A., Sahl H.-G. (2006). The co-evolution of host cationic antimicrobial peptides and microbial resistance. Nat. Rev. Microbiol..

[6] Hurdle J., O’Neill A., Lee R. (2012). Targeting bacterial membrane function: An underexploited mechanism for treating persistent infections. Nat. Rev. Microbiol..

[7] Zanetti M., Gennaro R., Skerlavaj B., Tomasinsig L., Circo R. (2002). Cathelicidin Peptides as Candidates for a Novel Class of Antimicrobials. Curr. Pharm. Des..

[8] Strempel N., Strehmel J., Overhage J. (2015). Potential application of antimicrobial peptides in the treatment of bacterial biofilm infections. Curr. Pharm. Des..

[9] Oren Z., Shai Y. (1998). Mode of action of linear amphipathic α-helical antimicrobial peptides. Biopolymers.

[10] Matsuzaki K. (1998). Magainins as paradigm for the mode of action of pore forming polypeptides. Biochim. Biophys. Acta—Rev. Biomembr..

[11] Huang H.W., Charron N.E. (2017). Understanding membrane-active antimicrobial peptides. Q. Rev. Biophys..

[12] Silhavy T.J., Kahne D., Walker S. (2010). The Bacterial Cell Envelope. Cold Spring Harb. Perspect. Biol..

[13] Malanovic N., Lohner K. (2016). Antimicrobial Peptides Targeting Gram-Positive Bacteria. Pharmaceuticals.

[14] Strahl H., Errington J. (2017). Bacterial Membranes: Structure, Domains, and Function. Annu. Rev. Microbiol..

[15] Yeaman M.R., Yount N.Y. (2003). Mechanisms of Antimicrobial Peptide Action and Resistance. Pharmacol. Rev..

[16] Wimley W.C., Hristova K. (2011). Antimicrobial Peptides: Successes, Challenges and Unanswered Questions. J. Membr. Biol..

[17] Sochacki K.A., Barns K.J., Bucki R., Weisshaar J.C. (2011). Real-time attack on single *Escherichia coli* cells by the human antimicrobial peptide LL-37. Proc. Natl. Acad. Sci. USA.

[18] Simpson B.W., Trent M.S. (2019). Pushing the envelope: LPS modifications and their consequences. Nat. Rev. Microbiol..

[19] Delcour A.H. (2009). Outer membrane permeability and antibiotic resistance. Biochim. Biophys. Acta-Proteins Proteom..

[20] Weidenmaier C., Peschel A. (2008). Teichoic acids and related cell-wall glycopolymers in Gram-positive physiology and host interactions. Nat. Rev. Microbiol..

[21] Simpson B.W., May J.M., Sherman D.J., Kahne D., Ruiz N. (2015). Lipopolysaccharide transport to the cell surface: Biosynthesis and extraction from the inner membrane. Philos. Trans. R. Soc. B Biol. Sci..

[22] May J.M., Sherman D.J., Simpson B.W., Ruiz N., Kahne D. (2015). Lipopolysaccharide transport to the cell surface: Periplasmic transport and assembly into the outer membrane. Philos. Trans. R. Soc. B Biol. Sci..

[23] Müller A., Wenzel M., Strahl H., Grein F., Saaki T.N.V., Kohl B., Siersma T., Bandow J.E., Sahl H.-G., Schneider T. (2016). Daptomycin inhibits cell envelope synthesis by interfering with fluid membrane microdomains. Proc. Natl. Acad. Sci. USA.

[24] Brogden K.A. (2005). Antimicrobial peptides: Pore formers or metabolic inhibitors in bacteria?. Nat. Rev. Microbiol..

[25] Nguyen L.T., Haney E.F., Vogel H.J. (2011). The expanding scope of antimicrobial peptide structures and their modes of action. Trends Biotechnol..

[26] Sträuber H., Müller S. (2010). Viability states of bacteria-Specific mechanisms of selected probes. Cytom. Part. A.

[27] Roth B.L., Poot M., Yue S.T., Millard P.J. (1997). Bacterial viability and antibiotic susceptibility testing with SYTOX green nucleic acid stain. Appl. Environ. Microbiol..

[28] Silverman J.A., Perlmutter N.G., Howard M., Shapiro H.M. (2003). Correlation of daptomycin bactericidal activity and membrane depolarization in *Staphylococcus aureus*. Antimicrob. Agents Chemother..

[29] Farha M.A., Verschoor C.P., Bowdish D., Brown E.D. (2013). Collapsing the proton motive force to identify synergistic combinations against *Staphylococcus aureus*. Chem. Biol..

[30] Epps D.E., Wolfe M.L., Groppi V. (1994). Characterization of the steady-state and dynamic fluorescence properties of the potential-sensitive dye bis-(1,3-dibutylbarbituric acid)trimethine oxonol (Dibac4(3)) in model systems and cells. Chem. Phys. Lipids.

[31] Shapiro H.M. (2000). Membrane Potential Estimation by Flow Cytometry. Methods.

[32] Wu M., Maier E., Benz R., Hancock R.E.W. (1999). Mechanism of Interaction of Different Classes of Cationic Antimicrobial Peptides with Planar Bilayers and with the Cytoplasmic Membrane of *Escherichia coli* †. Biochemistry.

[33] Waggoner A. (1976). Optical probes of membrane potential. J. Membr. Biol..

[34] te Winkel J.D., Gray D.A., Seistrup K.H., Hamoen L.W., Strahl H. (2016). Analysis of Antimicrobial-Triggered Membrane Depolarization Using Voltage Sensitive Dyes. Front. Cell Dev. Biol..

[35] Sims P.J., Waggoner A.S., Wang C.-H., Hoffman J.F. (1974). Studies on the Mechanism by which cyanine dyes measure membrane potential in red blood cells and phosphatidylcholine vesicles. Biochemistry.

[36] Cabrini G., Verkman A.S. (1986). Potential-sensitive response mechanism of diS-C3-(5) in biological membranes. J. Membr. Biol..

[37] Mason D.J., Lopéz-Amorós R., Allman R., Stark J.M., Lloyd D. (1995). The ability of membrane potential dyes and calcafluor white to distinguish between viable and non-viable bacteria. J. Appl. Bacteriol..

[38] López-Amorós R., Comas J., Vives-Rego J. (1995). Flow cytometric assessment of *Escherichia coli* and *Salmonella typhimurium* starvation-survival in seawater using rhodamine 123, propidium iodide, and oxonol. Appl. Environ. Microbiol..

[39] Ordóñez J.V., Wehman N.M. (1993). Rapid flow cytometric antibiotic susceptibility assay for *Staphylococcus aureus*. Cytometry.

[40] Gauthier C., St-Pierre Y., Villemur R. (2002). Rapid antimicrobial susceptibility testing of urinary tract isolates and samples by flow cytometry. J. Med. Microbiol..

[41] Hewitt C.J., Nebe-Von-Caron G. (2004). The Application of Multi-Parameter Flow Cytometry to Monitor Individual Microbial Cell Physiological State. Adv. Biochem. Eng. Biotechnol..

[42] Okuda K., Zendo T., Sugimoto S., Iwase T., Tajima A., Yamada S., Sonomoto K., Mizunoe Y. (2013). Effects of Bacteriocins on Methicillin-Resistant *Staphylococcus aureus* Biofilm. Antimicrob. Agents Chemother..

[43] Friedrich C.L., Moyles D., Beveridge T.J., Hancock R.E.W. (2000). Antibacterial Action of Structurally Diverse Cationic Peptides on Gram-Positive Bacteria. Antimicrob. Agents Chemother..

[44] Silvestro L., Weiser J.N., Axelsen P.H. (2000). Antibacterial and Antimembrane Activities of Cecropin A in *Escherichia coli*. Antimicrob. Agents Chemother..

[45] Morin N., Lanneluc I., Connil N., Cottenceau M., Pons A.M., Sablé S. (2011). Mechanism of Bactericidal Activity of Microcin L in *Escherichia coli* and *Salmonella enterica*. Antimicrob. Agents Chemother..

[46] Cheng M., Huang J.X., Ramu S., Butler M.S., Cooper M.A. (2014). Ramoplanin at Bactericidal Concentrations Induces Bacterial Membrane Depolarization in *Staphylococcus aureus*. Antimicrob. Agents Chemother..

[47] Clementi E.A., Marks L.R., Roche-Håkansson H., Håkansson A.P. (2014). Monitoring changes in membrane polarity, membrane integrity, and intracellular ion concentrations in *Streptococcus pneumoniae* using fluorescent dyes. J. Vis. Exp..

[48] (2017). World Health Organization Prioritization of Pathogens to Guide Discovery, Research and Development of New Antibiotics for Drug Resistant Bacterial Infections, Including Tuberculosis. https://www.who.int/medicines/areas/rational_use/PPLreport_2017_09_19.pdf?ua=1.

[49] Sabaté Brescó M., Harris L.G., Thompson K., Stanic B., Morgenstern M., O’Mahony L., Richards R.G., Moriarty T.F. (2017). Pathogenic Mechanisms and Host Interactions in *Staphylococcus epidermidis* Device-Related Infection. Front. Microbiol..

[50] Kelkar D.A., Chattopadhyay A. (2007). The gramicidin ion channel: A model membrane protein. Biochim. Biophys. Acta - Biomembr..

[51] Raghuraman H., Chattopadhyay A. (2007). Melittin: A Membrane-active Peptide with Diverse Functions. Biosci. Rep..

[52] Arndt-Jovin D.J., Jovin T.M. (1989). Chapter 16 Fluorescence Labeling and Microscopy of DNA. Methods Cell Biol..

[53] Johnson I., Spence M.T.Z. (2010). Chapter 22–Probes for Membrane Potential. The Molecular Probes Handbook. A Guide to Fluorescent Probes and Labeling Technologies.

[54] Johnson I., Spence M.T.Z. (2010). Chapter 8—Nucleic Acid Detection and Analysis. The Molecular Probes Handbook. A guide to fluorescent probes and labeling technologies.

[55] Boman H.G., Steiner H. (1981). Humoral Immunity in Cecropia Pupae. Curr. Top. Microbiol. Immunol..

[56] Berkowitz B.A., Bevins C.L., Zasloff M.A. (1990). Magainins: A new family of membrane-active host defense peptides. Biochem. Pharmacol..

[57] Bechinger B. (1997). Structure and Functions of Channel-Forming Peptides: Magainins, Cecropins, Melittin and Alamethicin. J. Membr. Biol..

[58] Dubos R.J., Hotchkiss R.D. (1941). The Production of Bactericidal Substances by Aerobic Sporulating Bacilli. J. Exp. Med..

[59] Yang X., Yousef A.E. (2018). Antimicrobial peptides produced by *Brevibacillus* spp.: Structure, classification and bioactivity: A mini review. Worldj. Microbiol. Biotechnol..

[60] Hartmann M., Berditsch M., Hawecker J., Ardakani M.F., Gerthsen D., Ulrich A.S. (2010). Damage of the Bacterial Cell Envelope by Antimicrobial Peptides Gramicidin S and PGLa as Revealed by Transmission and Scanning Electron Microscopy. Antimicrob. Agents Chemother..

[61] Rončević T., Krce L., Gerdol M., Pacor S., Benincasa M., Guida F., Aviani I., Čikeš-Čulić V., Pallavicini A., Maravić A. (2019). Membrane-active antimicrobial peptide identified in *Rana arvalis* by targeted DNA sequencing. Biochim. Biophys. Acta-Biomembr..

[62] Yasir M., Dutta D., Willcox M.D.P. (2019). Comparative mode of action of the antimicrobial peptide melimine and its derivative Mel4 against *Pseudomonas aeruginosa*. Sci. Rep..

[63] Virta M., Lineri S., Kankaanpää P., Karp M., Peltonen K., Nuutila J., Lilius E.M. (1998). Determination of complement-mediated killing of bacteria by viability staining and bioluminescence. Appl. Environ. Microbiol..

[64] Boulos L., Prévost M., Barbeau B., Coallier J., Desjardins R. (1999). LIVE/DEAD^®^ BacLight™: Application of a new rapid staining method for direct enumeration of viable and total bacteria in drinking water. J. Microbiol. Methods.

[65] Herrera G., Martinez A., Blanco M., O’Connor J.-E. (2002). Assessment of *Escherichia coli* B with enhanced permeability to fluorochromes for flow cytometric assays of bacterial cell function. Cytometry.

[66] Wu G., Wu H., Li L., Fan X., Ding J., Li X., Xi T., Shen Z. (2010). Membrane aggregation and perturbation induced by antimicrobial peptide of S-thanatin. Biochem. Biophys. Res. Commun..

[67] D’Este F., Benincasa M., Cannone G., Furlan M., Scarsini M., Volpatti D., Gennaro R., Tossi A., Skerlavaj B., Scocchi M. (2016). Antimicrobial and host cell-directed activities of Gly/Ser-rich peptides from salmonid cathelicidins. Fish. Shellfish Immunol..

[68] Huang J., Hao D., Chen Y., Xu Y., Tan J., Huang Y., Li F., Chen Y. (2011). Inhibitory effects and mechanisms of physiological conditions on the activity of enantiomeric forms of an α-helical antibacterial peptide against bacteria. Peptides.

[69] D’Este F., Oro D., Boix-Lemonche G., Tossi A., Skerlavaj B. (2017). Evaluation of free or anchored antimicrobial peptides as candidates for the prevention of orthopaedic device-related infections. J. Pept. Sci..

[70] Smart O.S., Goodfellow J.M., Wallace B.A. (1993). The pore dimensions of gramicidin A. Biophys. J..

[71] Lee E.K., Kim Y.-C., Nan Y.H., Shin S.Y. (2011). Cell selectivity, mechanism of action and LPS-neutralizing activity of bovine myeloid antimicrobial peptide-18 (BMAP-18) and its analogs. Peptides.

[72] Shang D., Sun Y., Wang C., Wei S., Ma L., Sun L. (2012). Membrane interaction and antibacterial properties of chensinin-1, an antimicrobial peptide with atypical structural features from the skin of *Rana chensinensis*. Appl. Microbiol. Biotechnol..

[73] Madhuri, Shireen T., Venugopal S.K., Ghosh D., Gadepalli R., Dhawan B., Mukhopadhyay K. (2009). In vitro antimicrobial activity of alpha-melanocyte stimulating hormone against major human pathogen *Staphylococcus aureus*. Peptides.

[74] Gee M.L., Burton M., Grevis-James A., Hossain M.A., McArthur S., Palombo E.A., Wade J.D., Clayton A.H.A. (2013). Imaging the action of antimicrobial peptides on living bacterial cells. Sci. Rep..

[75] Faust J.E., Yang P.-Y., Huang H.W. (2017). Action of Antimicrobial Peptides on Bacterial and Lipid Membranes: A Direct Comparison. Biophys. J..

[76] Akbari R., Hakemi Vala M., Hashemi A., Aghazadeh H., Sabatier J.-M., Pooshang Bagheri K. (2018). Action mechanism of melittin-derived antimicrobial peptides, MDP1 and MDP2, de novo designed against multidrug resistant bacteria. Amino Acids.

[77] Ladokhin A.S., Selsted M.E., White S.H. (1997). Sizing membrane pores in lipid vesicles by leakage of co-encapsulated markers: Pore formation by melittin. Biophys. J..

