# Supplementary material for: A Rapid Fluorescence-Based Microplate Assay to Investigate the Interaction of Membrane Active Antimicrobial Peptides with Whole Gram-Positive Bacteria
Source: Antibiotics (Basel). 2020 Feb 19;9(2):92. doi: 10.3390/antibiotics9020092 (PMC7168298; doi:10.3390/antibiotics9020092)
Supplement: Supplementary file 1 [file antibiotics-09-00092-s001.pdf]

Supplementary Materials

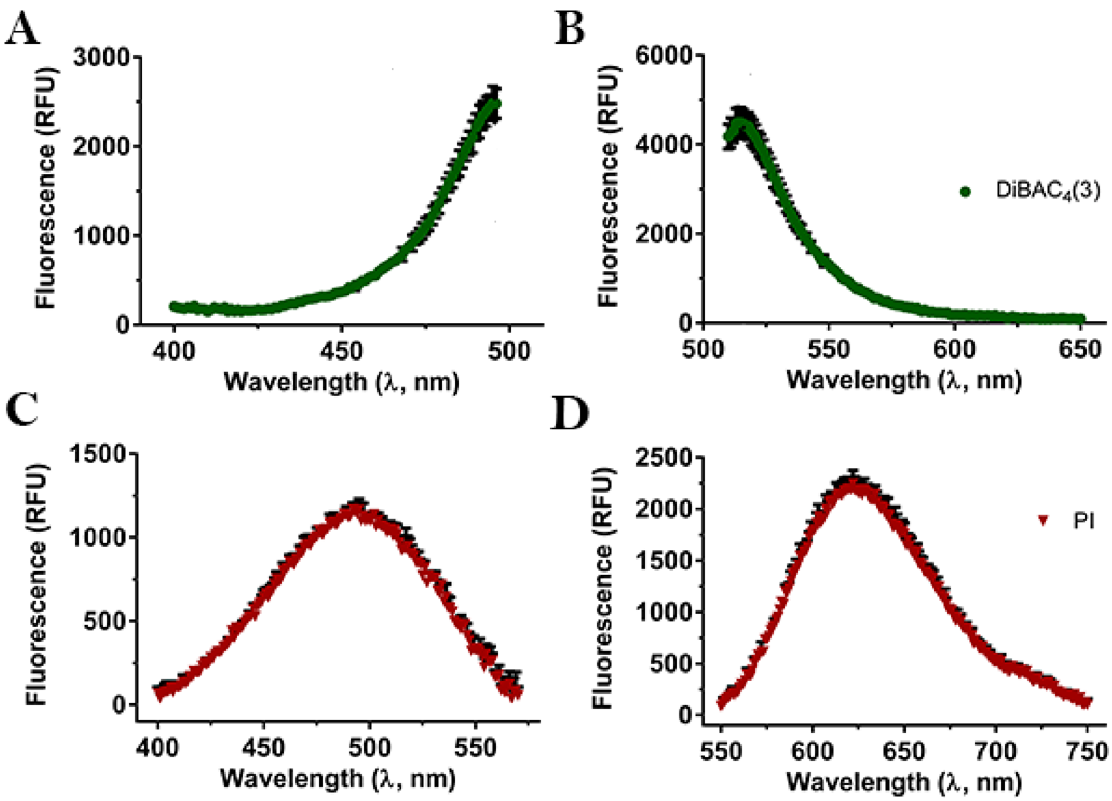

Figure S1. Separate fluorescence Excitation (**A, C**) and Emission (**B, D**) spectra of bis-(1,3-dibutylbarbituric acid)trimethine oxonol (DiBAC$_4$(3)) (**A–B**) and propidium iodide (PI) (**C–D**) in phosphate-buffered saline supplemented with 25 mM glucose (PBS-glc). (**A, C**) Excitation spectra of 500 nM DiBAC$_4$(3) with 516 nm (**A**) and 20 µg/mL PI with 617 nm (**C**) detection wavelengths. (**B, D**) Emission spectra of 500 nM DiBAC$_4$(3) with 496 nm (**B**) and 20 µg/mL PI with 490 nm (**D**) excitation wavelengths. For technical reasons due to instrument settings, spectra could not be measured at wavelengths > 496 nm (**A**) and < 510 nm (**B**).

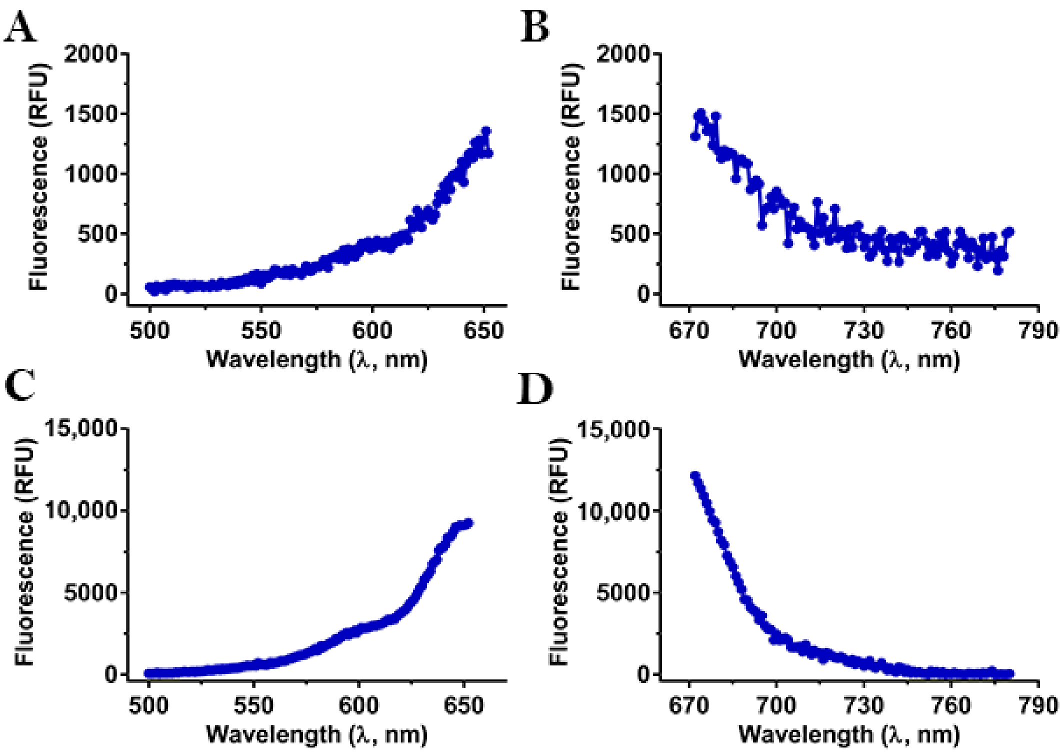

Figure S2. Fluorescence Excitation and Emission spectra of 400 nM 3,3'-dipropylthiadicarbocyanine Iodide (diSC$_3$(5)) in black wellplates (**A–B**) and low-binding OptiPlate (**C–D**). (**A, C**) Excitation spectra with 672 nm detection wavelength, and (**B, D**) emission spectra with 652 nm excitation wavelength (error bars were omitted for clarity). For technical reasons due to instrument settings, spectra could not be measured at wavelengths > 650 nm (**A, C**) and < 670 nm (**B, D**).

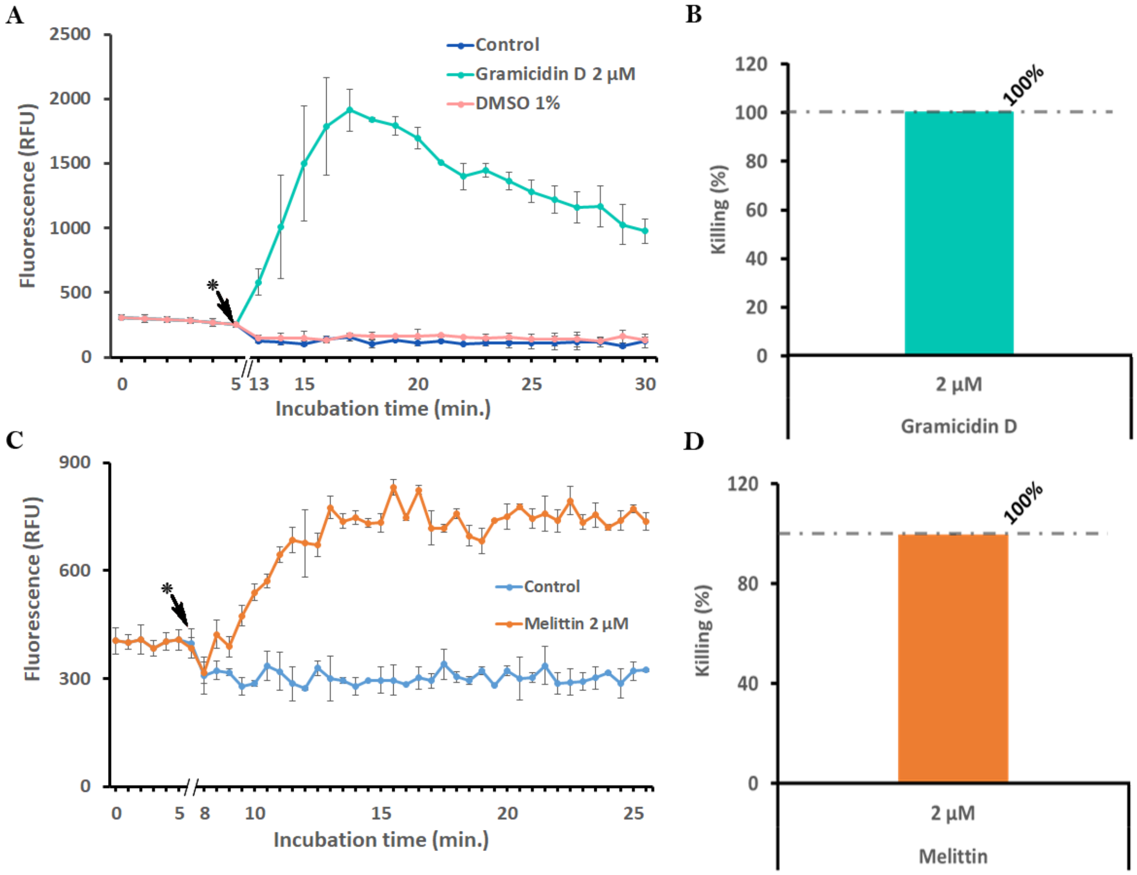

Figure S3. Membrane depolarization (diSC$_3$(5)) (**A**) and Killing (**B**) of *S. epidermidis* ATCC 35984 caused by gramicidin D and membrane permeabilization (PI) (**C**) and Killing (**D**) of *E.coli* ATCC 25922 caused by melittin. Experiments were performed with 10^7 CFU/mL of the indicated strains in PBS-glc with 400 nM diSC$_3$(5) (λ_{ex} = 652 nm, λ_{em} = 672 nm) and 5 μg/mL PI (λ_{ex} = 535 nm, λ_{em} = 617 nm) at 37°C. CFU counts were determined at 30 min incubation. (*) peptide addition.
